# Are cash transfers the panacea to local involvement in humanitarian decision-making? Evidence from World Vision projects in Umzingwane

**DOI:** 10.4102/jamba.v11i1.486

**Published:** 2019-02-21

**Authors:** Thabo Ndlovu, Siphathisiwe Ndlovu

**Affiliations:** 1 Institute of Development Studies, National University of Science and Technology, Zimbabwe; 2 Food and Income Security and Emergency Response, El Nino Projects, Zimbabwe Project Trust, Zimbabwe

## Abstract

Cash transfers are increasingly becoming a preferred mode of alleviating human suffering particularly in drought-affected communities in sub-Saharan Africa. The transition from conditional to unconditional cash transfer programming highlights strides that seek to promote local involvement and accommodate consumption choices of vulnerable recipients. This article sought to ascertain ways through which vulnerable societies’ voice and influence are magnified in unconditional cash transfer programming. In addition, the benefits of cash transfers and its contribution in building drought resilience of communities benefitting from World Vision interventions in Umzingwane District were explored. Through the descriptive survey design, data was collected using structured and unstructured questionnaires. Study participants were chosen using simple random and purposive sampling techniques. The findings showed that unconditional cash projects are beneficial in allowing recipients to prioritise deployment of scarce financial resources, influenced entrepreneurship within the locality and manipulated social capital. The article contends that building local resilience to drought was not feasible because of low cash allocations, which were inadequate to sustain household food needs, and this offered less space to invest in non-food items that support resilience processes. The article concluded that life-saving assistance including unconditional cash programming limit vulnerable populations from voicing their aspirations and influencing critical project decisions that have the potential to transform their resilience to drought, hence the need to deepen local participation in humanitarian and developmental projects.

## Introduction

Cash may be disbursed conditionally or unconditionally to selected project recipients (Walker et al. 2013). The unconditional transfer of cash entails not setting parameters to comply with prior to receiving resources, while conditional cash resources require beneficiaries to satisfy certain requirements before or after cash is made available. Unconditional cash programming has become one of the common ways through which resources are transferred to disadvantaged societies in sub-Saharan Africa (Gaarder 2012). International donor agencies such as Department for International Development (DFID) have played an extensive role in propagating the unconditional cash programming concept as it offers space to recipients to make choices on the deployment of their allocations (Gaarder 2012). Unconditional cash transfers do not bind benefitting households to given expenditures; however, they offer recipients an option to purchase deficit items elsewhere (Cunha 2014). In this context, ‘vulnerable group’ means individuals incapacitated to protect their interest because of limited options to meet household food requirements. To minimise vulnerable situations through humanitarian efforts, unconditional programming has become critical in creating space for disadvantaged households to participate in the design and implementation of projects. Not only does this process break social inclusion barriers, it has become a vital link for food-insecure households to select options compatible to their situation to bolster resilience against drought.

Devereux and McGregor (2014) argue that vulnerability can be lessened using cash transfers but cannot be dealt with, meaning distinct strategies to enhance inclusion and empowerment are likely to be more useful than providing cash. Barrientos, Hanlon and Hulme (2010) and Arnold, Conway and Greenslade (2011) suggest that there is a connection between cash transfers and vulnerability reduction as regular and predictable unconditional cash transfers overtime have the potential to aid defenceless households to contain drought impacts and invest in critical household livelihoods. One wonders how unconditional transfers in the humanitarian context respond to an assertion by Drucza (2016:3) that ‘pathways out of vulnerability are more complex than just in terms of income’. Sen (2001) supports that it is what communities do and are able to do with money that is vital, implying that no matter how small the amount may be, the way cash is deployed may provide the leverage out of vulnerability.

Social inclusion is the removal of social barriers and encouragement of incentives to promote access of diverse individuals and groups to development projects (Wortel 2009). Unpacking inclusion helps to devise avenues through which vulnerable groups could participate in the administration of unconditionally distributed resources. Answers were sought as to the role of unconditional cash transfer recipients and as to whether propping up their voices in programming does contribute to improved humanitarian responses and building of resilience to future droughts in Umzingwane District. The limited sources of income of vulnerable groups stifle their voice, representation and ability to uphold participation in social and cultural events (Kabeer 2010). Findings from a study conducted in Zimbabwe, Malawi, Ethiopia and Ghana by Rose et al. (2013) and the FAO (2013) depict that cash programmes enhanced social capital by permitting recipients to re-enter social networks and that they were viewed as equal partners with a right to influence outcomes.

According to Barnet and Weiss (2008) humanitarian support is deemed as any act intended to save lives and alleviate affliction, impartially, independently and neutrally providing aid urgently because of a disaster. The benefits of involving susceptible communities include fortifying the legitimacy and accountability of development institutions (Cornwall 2008; Creasy 2007) as well as enhancing social cohesion (Foot 2009). An atmosphere of excitement normally engulfs areas receiving cash as recipients exchange ideas when collecting money, strengthening cohesion. In addition, recipients discern village events and ultimately improve their participation in local social encounters (Drucza 2016).

Participatory approaches are encouraged in designing and implementation of aid to diffuse limitations of conditional transfers (Samson & Kaniki 2008). Arnstein (1969) describes participation as a course of redistributing power to permit the marginalised space to deliberately influence developmental decisions. The Ladder of Citizen Participation by Arnstein (1969) comprises hierarchical orders, namely non-participation (vulnerable communities are not involved in decision-making), tokenism (vulnerable groups are involved as a formality with the project owners having the ultimate say) and citizen control (vulnerable groups take control of decision-making). These gradations were examined to ascertain the extent of the influence of locals in the administration of unconditional cash programmes. The power to decide in designing and executing life-saving programmes is vital, though Pini and McKenzie (2006) view participation as a process that wastes time and resources without paying attention to its potential to enhance efficiency and effectiveness of aid. Cooke and Kothari (2001) support that the presence of external players tends to sway decisions away from the vulnerable, given the significant power they possess. He also cautions humanitarian response and recovery institutions against using participation as a tool for implementing preplanned projects.

Freire (1995) posits that the poor are creative and capable with a right to claim their rightful place as shapers of development initiatives. In support, Chambers (1997) avers that people-centred development acknowledges and emphasises that communities are shapers of their own destiny and that social capital ought to be managed to influence local drought mitigation through local networks. Observing their views is critical in comprehending the perceptions of the affected and informing of the value a community attaches to the challenges that confront it. This is consistent with Oliver et al. (2006) notion that participation enhances feelings of control, meaning and connectedness and that it contributes to building resilience and competencies in people as well as supporting several developmental processes. Molyneux, Jones and Samuels (2016) posit that participation by recipients should not be limited to certain project stages but should be wholly embraced because it is key in enhancing responsiveness to local concerns by development institutions. The role of participation is vital as espoused in the Rio Declaration on Environment and Development as one of the key enablers for decreasing vulnerability and enhancing resilience development (UN 1992). Despite the praise on the value of involving disadvantaged communities in problem-solving, O’Faircheallaigh (2010) argues that it generates complexity to humanitarian projects for minimal gains. The value of the target population to the overall humanitarian decision may be influenced by programme planners, who may confine recipients’ roles to certain project activities (Stevens, Berke & Song 2010). Such a practice puts a cap on locals’ potential to contribute and hinders perfection in performing a task.

Sen (2001:14) hints that ‘benefits meant exclusively for the poor often end up being poor benefits’. The involvement of vulnerable households in community projects not only presents space to influence their destiny but represents an act of learning-by-doing to sharpen their skills to deal with similar circumstances in future (Laesse 2010). Through participatory processes, the have-nots (disadvantaged), also considered economically excluded, may become part of determining relevant knowledge dissemination means, setting project goals as well as allocating critical resources. The coming together of communities during implementation of unconditional cash transfers plays a key role in social change and may result in means that community members would not arrive at working alone (Putnam 2002).

Resilience was examined through the lens of the 1973 resilience theory by Holling, in the field of ecology. Holling (1973) defines ‘resilience’ as a measure of persistence of a system and its ability to absorb change and disturbance and still maintain the same level of function. In this study, resilience was conceived to mean the ability of vulnerable groups to cope with stresses (drought). Resilience expressed the ability to transform so as to retain the same level of functioning as well as the aptitude to absorb shocks and bounce back better to deal with present and future disturbances. The theory depicts the strength of individuals or groups to persist and retain cohesiveness in face of persistent droughts. The transformability and innovativeness of the vulnerable groups to drought situations following the disbursement of unconditional resources put to test this concept. The question on whether unconditional cash transfers expose opportunities that recipients may exploit to accommodate future drought situations was explored. Folke (2006) contends that being resilient is not about being robust but utilising the chances resulting from disturbances.

### Background of the study area

Umzingwane District lies in Agro-ecological Regions IV and V, receiving less than 450 mm of rain annually. The insignificant amounts of rainfall subject the area to periodic seasonal droughts and compound the food security situation, which is on the decline in most areas of the district. The poor agricultural seasons characterised by late onset of erratic rainfall with frequent dry spells during critical stages of crop development limit dry land potential. Normally, harvest for this district last a maximum of 8 months, leaving communities with no choice but to supplement with local market purchases to meet household food deficits. The majority of communal farmers in this area favour maize over sorghum despite the prevalence rainfall conditions, which favour small grains unless complemented by irrigation. To mitigate drought, the Social Welfare Department has been distributing grain to affected households with World Vision International Zimbabwe complementing through unconditional cash transfers. The first phase of the unconditional cash programme was implemented between September 2015 and March 2016 for Umzingwane District, targeting food-insecure households in 14 wards.

## Methodology

The qualitative approach was used for the study to discern local views on unconditional cash transfers and their implications for social development. A descriptive survey design was employed to collect data using semi-structured questionnaires, face-to-face interviews and focus group discussions. The study targeted World Vision Zimbabwe unconditional cash recipients comprising mostly elderly people with limited survival options from the Mawabeni, Matshetsheni and Sibomvu areas.

Of the population of 345 unconditional cash beneficiaries from the target wards, 10% were randomly sampled to give each unit a chance of inclusion in the study. The sample size is consistent with Gray’s (2009) view that 10% is ideal to generate enough data to discern a phenomenon.

The unconditional cash recipients were randomly selected using World Vision beneficiary registers where recipients were assigned numbers, which were put in a container. The picking of numbers was random until the desired sample was achieved. The target group was informed of the day of the interview. The semi-structured questionnaire was administered by trained local extension workers and this helped improve response rates as all participants were available on the agreed days and time. Six face-to-face interviews were conducted with purposively chosen key informants, namely the village head, local chief, World Vision project officer, Social Welfare Department and rural district councils because of their vast knowledge and role in life-saving projects in the ward and district. Purposive sampling was used not only to select key informants knowledgeable about humanitarian projects but also to choose the study locations (Creswell & Plano Clark 2011). Permission was sought from the district administrator, rural district council, as well the traditional leadership to administer the semi-structured questionnaire, carry out face-to-face interviews and conduct three focus group discussions.

The collected data were largely qualitative; analysis of semi-structured instruments was done through the Statistical Package for the Social Sciences software to present results in pie charts and tables. Themes guided the processing of data collected though in-depth interviews to discern benefits of unconditional cash transfers and participation of recipients and to determine how resilience is influenced by such programming. Themes are defined as similar codes combined to generate units of meaning in the database (Creswell 2012). The study focused on matters respondents voiced most to formulate themes and these included issues that are unique and those with supporting evidence. Themes were interconnected into a storyline that unravelled unconditional cash transfers.

## Results and discussion

### Contribution of unconditional cash transfers

**FIGURE 1:**
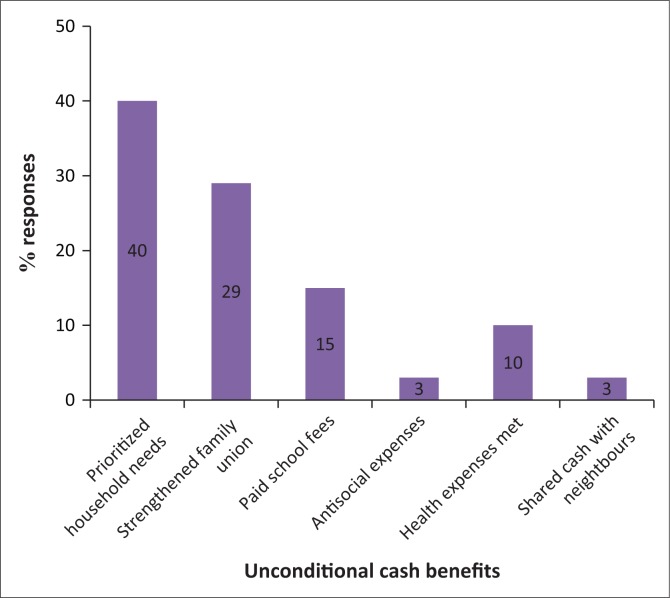
Benefits of receiving unconditional cash transfer.

The objective of the World Vision programme was to use cash as a modality of enhancing the decision-making abilities of drought-affected households and to assist in minimising decapitalisation in the short term. The examination of the output was done in the lens of the objectives of the programme as detailed in Figure 1.

In Figure 1, 40% indicated that unconditional cash transfers allowed them to prioritise household food items such as mealiemeal and cooking oil. This suggests that unconditional cash recipients channelled resources towards averting household food deficits, which was in line with the objectives of the project. Family union was enhanced as acknowledged by 29% of the respondents. Family cohesion was strengthened by predistribution training conducted by the implementing agency (World Vision Zimbabwe) and it encouraged collective budgeting at family level and supported the need to include women in decision-making. Trainings brought families together following the preprogramme implementation awareness on joint budgeting for households in benefitting wards. A significant number of 15% hinted that they paid school fees for their children, a sign that households managed to spread the payouts towards addressing pressing and essential needs at the time. Households receiving assistance for more than six members managed to reduce school fee debt but it was difficult for those with a membership of three or less as allocations were lower. The partial payment of school fees lessened the number of school dropouts, a situation ideal for a progressive society. Health expenses accounted for 10% of the respondents, with households having chronically ill members spending more. Antisocial activities and sharing cash with neighbours witnessed 3% each, respectively. The majority of the respondents did not share cash with neighbours.

The benefits of unconditional cash included its flexibility to purchase food items deemed convenient by recipients. Prioritisation of deficit items became possible, unlike other modes such as distributing food that rarely offer households choices to deploy resources to areas of greatest need. This view resonates with Cunha’s (2014) suggestion that unconditional cash transfers are flexible, as they do not tie benefitting households to given expenditures and are advantageous in situations where markets have failed, offering an option to purchase elsewhere. This prognosis has the strength to influence investments by the vulnerable groups to positively shift disaster thresholds (the point where response resources are no longer enough to deal with drought, beyond which negatives prevail) and enhance the local coping stamina to drought. The deployment of resources to improve access to education, health and family union were essential in providing the basic defence to any form of stress.

Joint budgeting between husbands and wives was instrumental in dissipating the notion that men are dictators on financial matters at household level and this helped defeat the potential to spend on antisocial activities. Silverlock (2010) confirms that participatory processes even at family level have the potential to generate trust, cohesion and redistribution of wealth through influencing expenditures on services most desired within a household. In-depth interviews conducted with a World Vision Zimbabwe project officer (June, 2016) point to the fact that preproject trainings on gender issues improved wife and husband relations as evidenced by no reported incidents of domestic violence following cash disbursements. A positive perception on gender roles at household level was created by engaging men as champions of gender transformation in patriarchal settings. The training exercises, however, did not completely diffuse the notion that allowing women to receive cash on behalf of the family compromised men’s ability to govern their households, as some felt wives would use cash to solely influence household decisions. There was minimal evidence to suggest that project recipients channelled part of the cash towards antisocial expenditure. Antisocial expenditure includes expenses that do not reduce household food deficits and at the same time do not improve the welfare of the family such as beer and cigarettes. This is consistent with a study by Tumusiime (2015) that disqualifies sentiments that communities may at times abuse relief resources, especially cash, by investing inconsiderably.

The local business witnessed an increase in the number of EcoCash agents (cashing out points) within the wards, which helped reduce distances travelled to access cash. The study could not at that point attribute the increase in EcoCash agents to unconditional cash programming only, given the increase in gold panning activities in the area. However, indications were that part of the objectives of establishing the cash payout points was to tap into the World Vision recipients market. Tumusiime (2015) supports the sentiment that demand for local products generally improves during the implementation of cash programmes and triggers the revival of local markets, which slightly creates minimal employment opportunities and the expansion of other livelihood sources. The major setback was that of low capital to meet cash needs of vulnerable groups, with some accessing their allocations late or in neighbouring areas.

The decision to adopt unconditional cash transfers was consistent with the 1994 International Federation of Red Cross and Red Crescent Societies and the International Committee of the Red Cross (ICRC) (World Bank 2013) that all humanitarians shall attempt to build disaster response using local capacities. The programme was pragmatic on the code of conduct as it helped in the provision of the much-needed investment for disadvantaged communities to stimulate economic growth and contribute to future drought resilience.

One of the respondents said in IsiNdebele, *ungamnika umuntu imali*, meaning you cannot give neighbours cash even if it is evident they have nothing to sustain themselves for that night. However, with food, it was easy to share; hence social capital was positively influenced. Sharing cash over food was a challenge given the difficulty of its mobilisation as it is culturally human for the latter to be distributed to neighbours in need. Retaining cash is advantageous because of its flexibility to deploy than food items. Intracommunity tensions because of jealousy between beneficiaries and non-beneficiaries were noted as recipients were allocated varying amounts of cash depending on the household size (Babajanian 2012; Miller, Tsoka & Reichert 2011). This eroded community cohesion, weakened social safety nets and the zeal to advance community developmental agenda collectively. Community consensus on the rightful people to benefit from cash programmes was compromised by conflicts arising from previous projects.

### Participation of recipients in unconditional cash programming

The preferred mode of distributing aid was through unconditional cash payouts and food items as accounted by 43% of respondents apiece. Unconditional cash transfers were favoured because of their flexibility, as recipients could spend on items beneficial to the household at the most convenient time. General food distribution presented fewer challenges for the elderly than cash, which was not easily accessible. The conflicting expectations on the mode of delivery of aid reflects poor local involvement in the entire programme, as such issues could have been noted during the project design stage.

**FIGURE 2:**
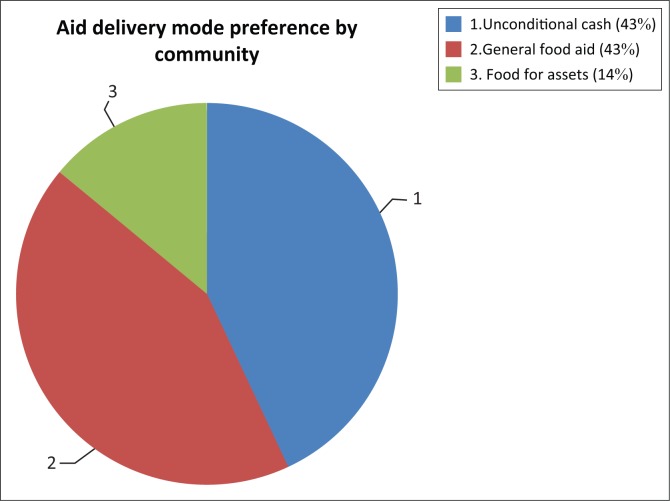
Community humanitarian distribution preferences.

The food for assets programme as a mode of distributing resources received the least support at 14%. The reason could be that the majority of recipients were elderly with limited strength to sustain food for assets demands. Figure 2 reveals that while recipients appreciated receiving unconditional cash benefits, they had more desire to receive food items as they felt that food distribution was fair and it built social capital as people could share more easily compared with cash. There were cases of misappropriation of cash as the majority of cash beneficiaries were elderly and could not operate sophisticated technologies; hence they tended to send younger people on their behalf. In some cases, these young people would not remit the expected amount. The other challenge was that cash withdrawal charges that reduced payouts were not clearly understood by the elderly. The results concur with Holmes’ (2013) and Adato, Roopnaraine and Becker’s (2011) propositions that involving beneficiaries and non-beneficiaries helps align cash programmes with local settings such as literacy levels and infrastructure development to sustain operationalisation. The findings question the recipients’ involvement in deciding the best way of channelling assistance without compromising on benefits because of vulnerable households.

Poor beneficiary consultation was further magnified by local preferences, which seemed to be in contrast with the mode of implementation adopted by the agency. While unconditional cash programming finally got the approval of the authorities, it was the vulnerable households that suffered the negatives of the project imbalance in terms of satisfying their expectations.

### Project beneficiary’s role in unconditional cash programming

The cash transfer intervention expected recipients to play a key role in its operationalisation. Recipients were anticipated to be instrumental in targeting deserving cases and to attend training sessions, though their contribution did not include the determination of cash transfer amounts. Figure 2 expresses the role played by recipients in implementing the cash programme.

**FIGURE 3:**
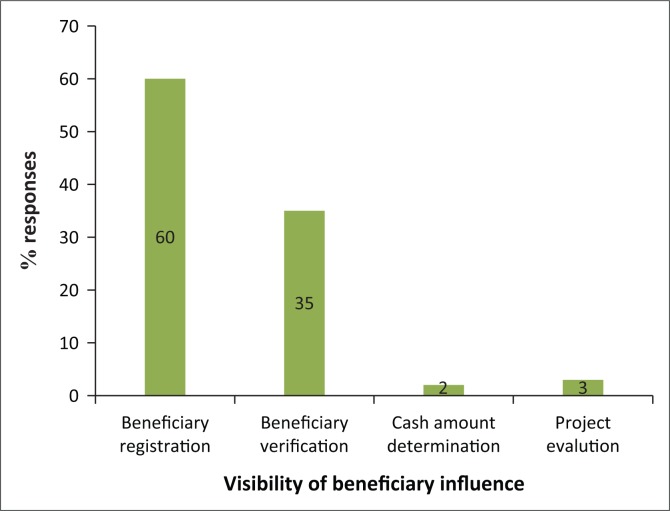
Recipients role in the project.

The recipients’ voice was significant during registration (targeting) as it accounted for 60% while 35% acknowledged that verification of programme recipients was conducted (Figure 3). This has become common with most relief programmes as they demand that recipients screen themselves for eligibility in accordance with defined criterion. Community-based targeting was the most commonly adopted process for selecting deserving household recipients, and the verification process was conducted by World Vision Zimbabwe representatives. The voice of unconditional cash beneficiaries was insignificant in project evaluation and cash payout value determination, with 3% and 2%, respectively.

**FIGURE 4:**
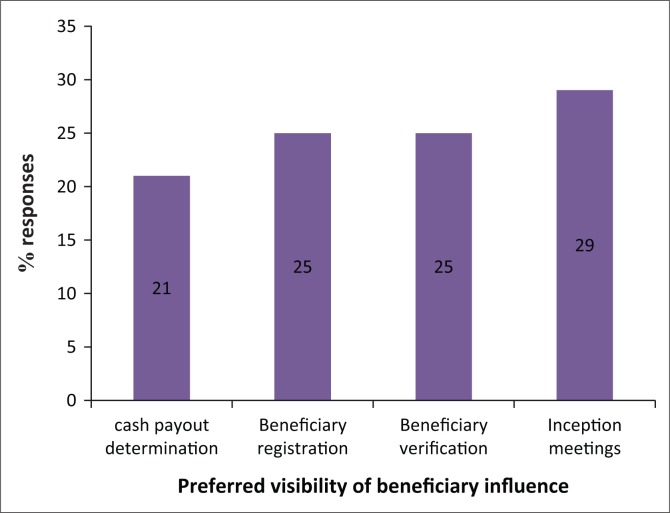
Preferred recipient roles in the project.

Figure 4 speaks to the unconditional cash programme activities, where beneficiaries preferred to be more active. The inception of the programme accounted for 29% of participant responses because of the strategic nature of the activity in defining local gaps in building resilience. Recipients believe this is a stage where funders can adjust ideas to embrace local perceptions. Beneficiary registration and verification activities were indicated by 25% of participant responses apiece, a sign that they treasured these activities because they understood household vulnerabilities better. The determination of the value of cash payouts was acknowledged by 21%. Recipients felt cash payouts were too small to meet basic household needs. The programmes advanced a minimum of $15.00 for households constituting three members and below while those with four and above were given an additional $5.00 for every additional member.

The participation of recipients was confined to the mobilisation for consultative meetings and identification of potential cash transfer recipients, which was overseen by village leaders. Such arrangements resemble a view by Arnstein (1969) that drought mitigation information for proposal development is provided to recipients at a later stage, too late to redesign the intervention to their benefit. The targeting process was not a smooth process as cases were noted of elite members capturing participatory processes to advance their interests on the pretext of pursuing those of the entire community. The elite are described as that group of influential less poor individuals such as progressive farmers and villager leaders who normally receive visitors and articulate local interest yet at the same time benefit more in terms of advice and services (Chambers 1994). The dearth in information on the unconditional cash transfers was possibly a ploy to interfere with effective targeting and encourage project capture by local elites and politically positioned members. Lack of transparency on targeting criteria contributes to exclusion of deserving cases (MacAuslan & Riemenschneider 2011). The implementing organisation was challenged by programme partners to increase awareness programmes so that deserving cases located in communication-constrained areas are mobilised timeously to participate, a view also shared by Ulrichs and Roelen (2012) that the rate of exclusion soars in highly inaccessible areas.

The failure by humanitarian organisations to involve vulnerable groups in designing the project may be an indication that they share a similar opinion to that of O’Faircheallaigh (2010), who sees the involvement of locals as an activity that does not add value to the entire assistance programming. The findings highlight the propagation of tokenism, as described by the Ladder of Citizen Participation. Tokenism entails development agencies’ deliberate intentions of involving locals as a ploy to authenticate predetermined project outcomes. The aspirations of recipients to increase cash transfer value was contrary to views by project implementers, who argued that the space for vulnerable groups to contribute is limited for such because of scarce financial resources. One wonders if communities should not have a say in what is theirs despite its value. The local ward councillor for the area in one of the interviews (June 2016) says, ‘yes the target group is vulnerable, failure to consult them on cash value is something else’. This opinion runs counter to the notion of some implementing partners that involving communities in determining how much they should receive is not a pragmatic process, as it is likely to attract undeserving cases whose presence could have derailed the entire programme. The other reason advanced was that scientific methods used to calculate individual food allocations and subsequent cash value were too sophisticated for an ordinary community member to discern, excluding recipients from negotiating what works for them.

While concerns were raised on the exclusion of deserving cases, those tasked with leading the process were accused of exploiting the opportunity to settle personal scores by deliberately ignoring genuine cases in preference to close and non-deserving relatives. The results are consistent with postulations made by Soares, Fábio Ribasand Osorio (2010) that despite targeting being a valuable practice in aiding effective deployment of resources, it is seen as a complex process that often excludes eligible food-insecure households.

The disparities in targeting of unconditional cash transfer beneficiaries emanate from the elite not disseminating information effectively and at the same time using their influence to vouch for the less deserving at the expense of the needy. In defence of the targeting process, one of the village heads (June 2016) said ‘at times it is a challenge to understand the vulnerable as they always have excuses’. This implied that the information was disseminated but at times the target group either attends meetings late or never attends at all; hence villagers exploit such opportunities.

### Drought resilience through cash transfer

Resilience building through the World Vision implemented cash transfer project was anchored on training of recipients to encourage effective deployment of cash resources. In addition to the provision of cash, recipients were exposed to drought risk reduction initiatives such as conservation agriculture, post-harvest management, household budgeting and nutrition matters. The idea was to transform households and embolden sound decision-making in addressing present and future drought effects. Drought resilience, not a fixed quality within households, is one of the longed-for outcomes that some community members perceive to describe their ability to successfully meet and surmount challenges and obstacles. This view is also supported by Folke et al. (2010) and Holling (1973) notion that it is a measure of the persistence of systems and of their ability to absorb change and disturbance and still maintain the same relationships’. The dimensions of community resilience may be characterised by community processes, resources and institutional organisations. In this context, the extent to which vulnerable groups used allocated cash to address identified areas of weaknesses reflects resilience resolve. The flexibility of unconditional cash transfer and subsequent investment choices exhibited by the recipients gave insight into how prepared they were for future droughts. The inadequacy of cash allocations, irregularity of disbursements and limited decision-making powers by the recipients on humanitarian interventions compromises the building of resilience to drought by depriving recipients of opportunities to invest in enterprises that offer competitive advantage against drought. The cash payouts were said to be too irregular, a scenario that made it extremely difficult to plan and make meaningful investments to ameliorate the effects of drought. Irregular payments deepened vulnerability among households as they triggered recipients to dispose of critical assets to meet food deficits. Arnold et al. (2011) concur that consistency of cash assistance overtime has the ability to influence livelihood diversification and build local capacities against drought. Only once day-to-day household food requirements are met can a community consider investing for tomorrow.

What also complicated resilience building was the fact that the cash payouts sustained recipients for a minimum period not exceeding 2 weeks, meaning they had to mobilise additional resources to meet food deficits before cash was next disbursed. Discussions with the ward councillor (June 2016) overwhelmingly supported the upward review of cash allocation to firstly allow recipients to meet monthly food needs without having to dispose of critical assets to meet monthly deficits. The ability to interact with colleagues presented opportunities to listen and advance suggestions on community matters and in the process social exclusion was dealt with. Social interactions resulting from unconditional projects were temporary as the cash allocations were not significant enough to permit a second or third round of purchases before receiving the next allocation.

One of the respondents said: ‘cash collections present an opportunity to interact with old friends and to be informed on happenings within the vicinity’ (June 2016), while findings by Vadapalli (2009) suggest that poor people feel humiliated and isolated because of failure to fulfil their social responsibility and partake in social events. The strengthening of social capital through unconditional cash transfers resonates with the third priority of the Sendai Framework for Disaster Risk Reduction 2015-2030 encourages public and private investment in structural and non-structural measures to promote social resilience of persons (UNISDR 2015). Social connectedness of drought-threatened communities provides society with the impetus to mobilise and pursue agendas that generate collective benefits.

While communities supported unconditional cash programming, they believed that it should be combined with other locally suited drought mitigation measures, especially on livestock, to curb losses for sustained drought recovery initiatives. Despite the fact that the unconditional cash transfer project was supported by software activities like training on post-harvesting management and conservation agriculture, little benefit was realised as most beneficiaries were less energetic and too old to conceptualise and successfully implement conservation agriculture. A local village head (June 2016) said that ‘the programme has created opportunities for self-employment’. This was evident as the number of cash payout agents were said to have increased, though results were not conclusive on the number. The benefits of unconditional cash programming spread beyond the life of the programme as established local pay agents will eliminate transport expenses and allow locals to withdraw cash at minimum costs.

## Conclusion

The participation and influence of vulnerable populations in humanitarian and development work has become a buzzword and a dream in contemporary drought resilience. While there is a view that participation generates minimal benefits to the entirety of programming and implementation, it should be noted that unless the benefitting community and development institutions collaborate during project design, implementation and evaluation, resource deployment shall continue to contribute less to ameliorate vulnerability. The equivocal decision presented by the recipients on the mode of distribution of relief assistance signifies a gap in the way programmes are designed to embrace local settings for adaptable interventions that enhance local capacities to handle future droughts. The paradox of local involvement is compounded by the disputed outcomes of the targeting process, as the community leadership was accused of failure to mobilise and preside over a fair selection process. Participation of recipients was limited and it perpetuated non-involvement and tokenism under the guise of advancing local interest, thereby robbing vulnerable individuals of potential gains in the entire programme. Slow onset hazards like drought normally provide warning and create space for recipients’ consultation on priority interventions, maximisation of resources in life-saving programmes and viable means of building resilience. The voice of the vulnerable in humanitarian decisions cannot be achieved by seeking authority to operate from local leaders, but through inclusive drought mitigation planning efforts, which aid locals to strategically position themselves against the hazard. The article was not conclusive on whether the benefits offered by unconditional cash programmes outweigh those presented by food relief; hence this area needs to be explored, including the gender dynamics presented by unconditional payouts.
